# Cases of Acute Rheumatism Cured by Lemon-Juice

**Published:** 1852-02

**Authors:** 


*Cases of Acute Rheumatism cured by Lemon-Juice.*—Under the care of Dr. Babington. In 1850, we directed the attention of our readers to a new and successful method of treating acute rheumatism, introduced by Dr. Owen Rees. The favorable results had been obtained by the administration of lemon-juice, and we then stated, "it appears that Dr. Rees' colleagues, Drs. Addison and Babington, who coincide with him in his views as to the efficacy of the remedy, have put the lemon-juice to the test, and found it extremely useful." Further trials have had the effect of establishing more and more the great value of lemon-juice as a therapeutical agent in acute rheumatism; and so convinced has Dr. Babington become of the value of this simple medicine, that we find him state, in the introductory lecture for this session, that Dr. Rees's new method of treating acute rheumatism held a prominent rank among the strides which have been made in the various departments of medical knowledge since his (Dr. Babington's) pupilage.

It is certainly with good reason that Dr. Babington passed this encomium, for he has prescribed the lemon-juice in numerous cases, and has found it answering the most sanguine expectations. Of a few of these cases we shall subjoin abstracts, in order to put our readers in possession of the facts upon which rests the reputation of the new remedy. We would, however, first mention some remarks of great importance which were made by Dr. Babington.

The lemon-juice which was employed was procured from a wholesale confectioner, the price being no more than sixpence per pint, when retailed in small quantity, and considerably less when furnished by the gallon.—When Dr. Babington commenced making trials of the lemon-juice, he prescribed it, according to the recommendation of Dr. Owen Rees, in doses of from one to two ounces, three times a day, for an adult. More recently, however, he had ordered not less than three ounces, and much more—usually six ounces—taken at a draught, and without any admixture, three times a day. It might be supposed that the patient would find some difficulty in drinking so large a quantity of such a sour liquid, but this is rarely the case; nor does it in general produce tormina, nor otherwise disagree with the alimentary canal.—Instead of relaxing the bowels, as might have been expected, it renders them somewhat costive, so that it not unfrequently becomes necessary to exhibit an aparient.—The juice produces no very decided effect upon the kidneys, merely tending by its quantity to promote their secretion. It increases cutaneous action, but to what extent it is difficult to determine, because in rheumatism, a disease in which this remedy is chiefly useful, the complaint itself is marked by the occurrence of profuse perspiration. The only unequivocal effect which uniformly takes place is a diminution in the number and power of the pulse, and of the heart's action. Whether it alters the character of the blood, or whether it affects the heart by diminishing nervous influence, Dr. Babington has not had an opportunity of determining.

We shall now shortly allude to four cases, which were treated with lemon-juice by Dr. Babington.

Case 1.—James D——, a tall and athletic young man, a dock laborer, twenty-two years of age, was admitted into Naaman's ward, August 20, 1851, with acute rheumatism. He has a white and coated tongue, profuse perspiratiou, thirst, a hard, full pulse at 92, and costive bowels. There are swellings in the knees and wrists, which are red and tender to the touch, and his debility is so great that he is scarcely able to walk to his bed.

The patient stated, on admission, that he had been ill three weeks, and that he had suffered from a similar attack eight years before, which lasted for one month.—There was no irregular sounds about the heart.

*Treatment.*—To take six ounces of lemon-juice three times a day; a drachm of compound rhubarb powder to be given on the morning following admission.

First day.—Bowels relieved, pulse 76, softer; patient has less pain in the joints; to continue the remedy.

Third day.—Pulse 70, soft; no pain or swelling of the joints. Patient is up and dressed; he declares himself quite well, and requests to be discharged. He is asked to remain a day or two longer to improve his general strength; but he declines, as he states that he is well able to work.

Case 2.—Samuel H——, aged nineteen, of slight form, a warehouseman, much exposed in his work to currents of air; never before had any similar attack. For four or five days he has had slight swelling and tenderness in the knees and ankles.

On admission into Naaman's ward, on September 10, 1851, there were great swelling, redness, and intense pain of the right elbow-joint, extending both upwards for several inches and downwards into the wrist. The tumefaction was so severe, that it was a question whether the inflammation was not rather phlegmonous than rheumatic, but the pain and swelling in the other joints settled this point. Tongue moist but white; pulse 116, full; bowels regular; skin perspirable. To take six ounces of lemon-juice three times a day; low diet.

First day.—Pulse 100, softer; bowels regular; arm and elbow-joint much the same as yesterday. To go on with the remedy.

Fifth day.—Pulse 88, soft; the pain and swelling of the elbow have decreased rapidly since the last visit, and are now very slight; bowels still regular.

Eighth day.—No pain anywhere, but still some stiffness of the right elbow-joint, which prevents the complete extension of the arm. Bowels regular.

Twelfth day.—Dismissed, free from all complaint, and having the full use of his right arm, the elbow-joint of which has returned to its natural size. The patient required one dose of the compound rhubarb powder during the treatment.

Case 3.—James S——, aged twenty, a baker, much exposed to heat and cold in working at night; never before had a similar attack; has suffered for ten days pain and swelling of both shoulder-joints. Admitted into Naaman's ward on September 10, 1851, having great pain and swelling of both shoulders, but especially the left, so that he is very reluctant to attempt to raise the left arm. Pulse 108, hard; skin hot; tongue white and dry, much febrile excitement; bowels confined. Ordered six ounces of lemon-juice three times a day; fifteen grains of compound extract of colocynth to be taken at night.

First day after admission.—Bowels well relieved; pulse 82; less pain; to continue the lemon-juice.

Fifth day.—Pulse 80; pain and swelling much abated; to go on.

Eighth day.—No pain or swelling; is able to raise both hands above his head, and so far as regards the rheumatism is well. Patient continues in the hospital in consequence of a swelling in the groin, which was opened two days after his admission, and is going on favorably. He required purgative medicine every alternate day whilst taking the lemon-juice.

Case 4.—John H——, aged twenty-two, single, a policeman, formerly a farm-laborer, with florid complexion, light hair, and strumous appearance; habits temperate.—The patient was attacked with acute rheumatism three years ago, but has since had good health, until within the last fortnight, when he began to suffer from his present illness. The inner sides of each foot and ankle were first affected, and then the elbows and hands became swollen, red, and painful, in which state he was admitted into Naaman's ward, No. 8, on July 9, 1851.

There was then a murmur heard on the heart's systole; pulse 104, hard, strong and incompressible; tongue coated and white; countenance congested; appetite gone; bowels costive; urine acid, specific gravity 1010, about one quart passed daily; perspiration profuse; pulse 92. Ordered six ounces of lemon juice, three times a day.

First day after admission.—The pain and swelling of the elbows and hands decreasing; ordered to continue the remedy.

Fifth day.—The lemon-juice seemed to cause a little griping pain in the bowels, which were moved twice a day whilst its use was continued. The pulse 80; small, weak, and compressible. There remained not a vestige of pain nor swelling anywhere.

Tenth day.—Pulse still 80; no *bruit* heard now with the systole of the heart. The patient has been quite free from all complaint for several days. The pulse retains the same character as on the fifth day. Discharged cured. —*Ibid.*

